# Immuno-protective response of Asian seabass (*Lates calcarifer*) to inactivated vaccines against *Streptococcus iniae* and *Vibrio harveyi*

**DOI:** 10.1186/s12917-024-03935-x

**Published:** 2024-03-08

**Authors:** Ahmad Erfanmanesh, Babak Beikzadeh, Majid Khanzadeh, Mojtaba Alishahi

**Affiliations:** 1 Animal Biological Product Research Group, Academic Center for Education, Culture and Research (ACECR), Tehran Organization, Tehran, Iran; 2 Department of Cell and Molecular Biology & Microbiology, Faculty of Biological Science and Technology, University of Isfahan, Isfahan, Iran; 3 Department of Fisheries, Faculty of Fisheries and Environmental Sciences, Gorgan University of Agricultural Sciences and Natural Resources, Gorgan, Iran; 4 Department of Clinical Sciences, Faculty of Veterinary Medicine, Shahid Chamran University of Ahvaz, Ahvaz, Iran; 5 Centre of Excellence for Warm Water Fish Health and Disease, Shahid Chamran University of Ahvaz, Ahvaz, Iran

**Keywords:** Asian seabass, Immune parameters, Streptococcosis, Vaccine, Vibriosis

## Abstract

**Background:**

In this study, the protective immunity and immunogenicity of the monovalent and bivalent *Streptococcus iniae* and *Vibrio harveyi* vaccine were evaluated in Asian seabass. To analyze immune responses, 1200 Asian seabass with an average weight of 132.6 ± 25.4 g were divided into eight treatments in triplicates (50 fish per tank) as follows: *S. iniae* immunized by injection (SI), *V. harveyi* immunized by injection (VI), bivalent *S. iniae* and *V. harveyi* (SVI) immunized by injection, *S. iniae* immunized by immersion (SIM), *V. harveyi* (VIM) immunized by immersion, bivalent *S. iniae* and *V. harvei* (SVIM) immunized by immersion, phosphate-buffered saline (PBS) by injection (PBSI) and control group without vaccine administration (CTRL). Blood and serum samples were taken at the end of the 30th and 60th days. Then the vaccinated groups were challenged with two bacteria (*S. iniae*) and (*V. harveyi*) separately and mortality was recorded for 14 days.

**Results:**

This study reveals that there is no significant difference in the hematological parameters on the 30th and 60th days of the experiment in the vaccine-immunized groups compared to the CTRL group (*P* > 0.05). Meanwhile, there was no significant difference in the amount of serum albumin level, respiratory burst activity, and serum bactericidal activity in the vaccine-immunized groups compared to the CTRL group on the 30th and 60th days of the experiment (*P* > 0.05). Total protein on the 60th day (in the VI and SVI groups), globulin on the 30th day (in the VI and SVI groups) and the 60th day (in the VI group) compared to the CTRL and PBSI groups had a significant increase (*P* < 0.05). Complement activity (in the VI and SVI groups) and lysozyme (in the SI and SVI groups) increased significantly compared to the control group (*P* < 0.05). Serum antibody titer against *S. iniae* had a significant increase in the SI, VI, SVI and SVIM groups compared to the CTRL and PBSI groups (*P* < 0.05). Serum antibody titer against *V. harveyi* had a significant increase in the groups immunized with the vaccine compared to the CTRL and PBSI groups (*P* < 0.05). A significant increase in the relative percentage survival (RPS) following challenge with *S. iniae* in the SVI (86.6%), SI (83.3%,) and VI (73.3%) groups were observed compared to the CTRL (43.3%) and PBSI (40%) groups (*P* < 0.05). Also, a significant increase in the RPS after challenge with *V. harveyi* in the SVI group, VI 86.6%, SVI 83.3%, VIM 80% and SVIM 76.6% were observed compared to the CTRL (46.6%) and PBSI (50%) groups (*P* < 0.05).

**Conclusion:**

Overall, the results demonstrated that the bivalent vaccine of *S. iniae* and *V. harvey*was able to produce significant immunogenicity and RPS in Asian seabass

## Background

The significant increase in population and the demand for aquatic consumption besides the limitation of natural resources have led to the development of the aquaculture industry in the world [1]. Aquaculture has recently emerged as one of the most efficient methods for producing food and has quickly developed into an active and expanding industry [2]. The need for aquatic proteins has led to the change of extensive farming systems towards intensive and super-intensive farming [3]. Therefore, intensive and super-intensive fish farming increases the susceptibility of fish to infectious diseases, which has a serious negative impact on the economy [4]. The most serious pathogens in aquaculture are bacteria (54.9%), viruses (22.6%), parasites (19.4%) and fungi (3.1%), respectively [5]. The main issue in the aquaculture industry is bacterial infections [6]. Antibiotics are used as the first choice to control bacterial disease [7]. However, the uncontrolled use of antibiotics disrupts animal protein production, microbiota, nutrition and immunity which poses a great risk to human health [8]. Antibiotics can eliminate beneficial microorganisms and disrupt the microbiota of the gastrointestinal tract, affecting nutrition and immunity [9]. Antibiotic usage has the potential to select resistant bacteria and transfer resistance genes to animals and human microbiome [10]. Due to the mentioned problems, the use of antibiotics is no longer a priority and there is a serious need to find suitable environmental alternatives to solve problems related to antibiotics. Today, vaccines are the best way to prevent and control infectious diseases [11]. Unlike antibiotics, vaccines stimulate the fish’s immune system and protect the fish from pathogens [12]. In other words, the significant increase in requests and the intensive system in aquaculture lead to the intensification of stress and the occurrence of diseases in aquatic animals [13]. The two most prevalent bacterial infections in Asian seabass farming are Vibriosis and Streptococcosis, which can cause severe economic losses [14]. Asian seabass infected with *Vibrio harveyi* (*V. harveyi*) shows lethargy, anorexia, abnormal swimming, abdominal distension, ulcerative skin lesions and darkened skin [15]. Meanwhile, fish infected with *Streptococcus iniae* (*S. iniae*) shows clinical symptoms such as irregular swimming, loss of equilibrium, unilateral or bilateral exophthalmia, darkening of the skin, darkening of the cornea, and hemorrhaging in the fins [16].

Monovalent vaccinations are currently no longer cost-effective due to co-infection rises in agriculture. On the other hand, polyvalent vaccines are vital in the aquaculture vaccination process because of stress reduction, cost-effectiveness, and resistance to multiple infections [6].

Recently, the demand for purchasing Asian seabass has increased. This could be due to the good taste and increasing awareness about the beneficial properties of this fish, such as protein and omega-3 fatty acids, which are high in Asian seabass [17]. Asian seabass (*Lates calcarifer*) is a euryhaline carnivorous fish that has high economic value, fast growth and can be widely cultured in freshwater and marine waters [18]. Asian seabass is a protandrous hermaphrodite and fish that has wide physiological tolerances [19].

Previous research showed that polyvalent vaccines have a positive and significant effect on the immunogenicity and survival of fish. In a study, the effect of polyvalent vaccine against Streptococcosis, Vibriosis, and Aeromonas Septicemia in *L. calcarifer* was investigated and it was reported that the vaccine has a significant effect on the immunogenicity of Asian seabass [14]. Moreover, in another study, the effect of polyvalent yersiniosis, streptococcosis and lactococcosis, vaccine was investigated in *Oncorhynchus mykiss* and it was reported that this vaccine has significant immunogenicity in rainbow trout [6]. Considering the positive effects of vaccines in previous studies, in this study the effectiveness of bivalent and monovalent vaccines by immersion and injection method on immune response and disease resistance against Streptococcus and Vibriosis in *L. calcarifer* were investigated.

## Results

### Hematological parameters

**Fig. 1:**
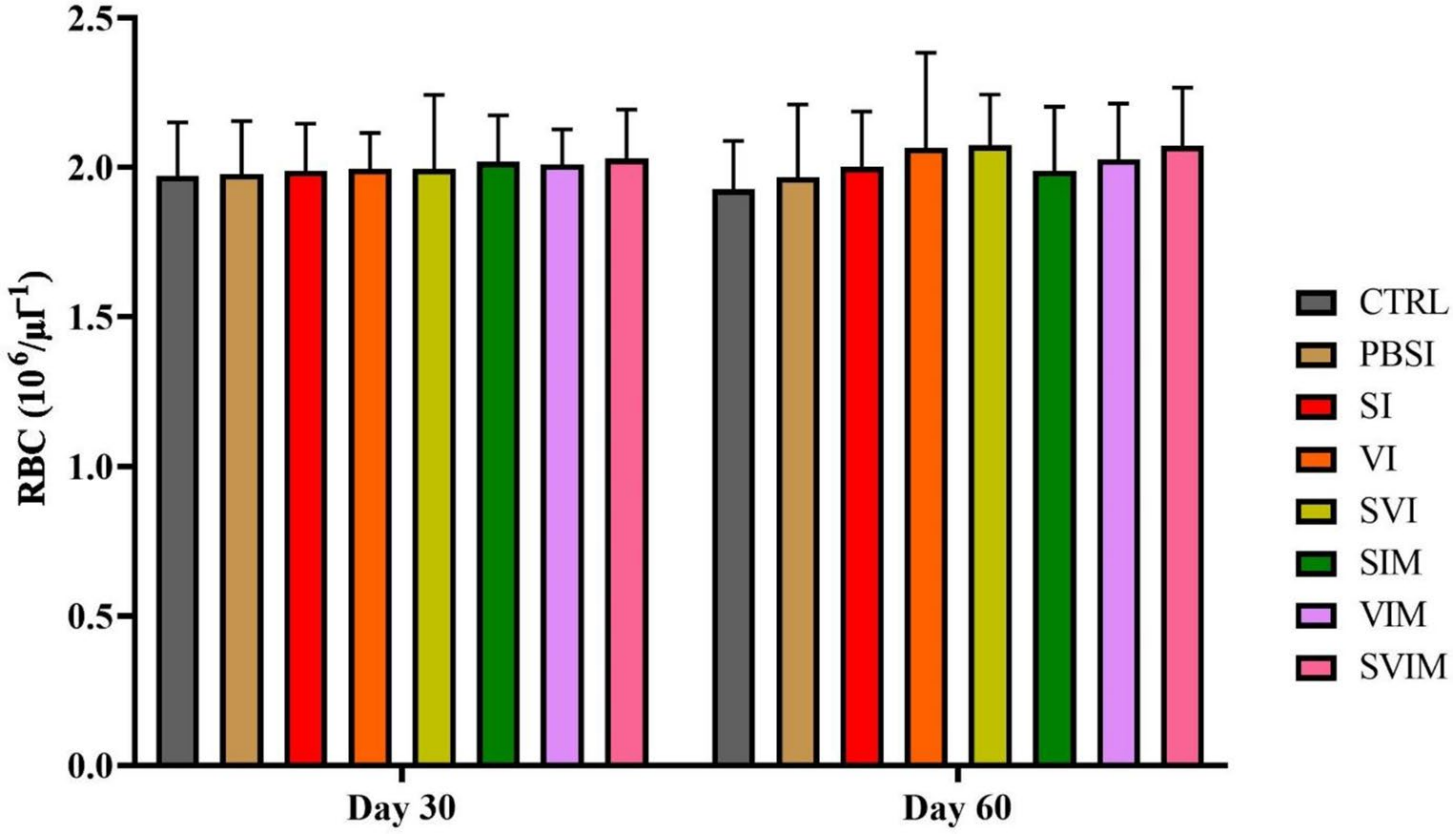
RBCs count in the groups vaccinated with *S. iniae* and *V. harveyi* in Asian seabass. The effect of time in all groups were not significant (*P* > 0.05). Interaction of time × treatment: *P* = 0.9909. Values are Mean ± SD (*n* = 9 fish per treatment). Statistical significance was measured using Tukey’s test and two-way ANOVA to show the difference groups over time. RBC: red blood cell, CTRL: control, PBSI: Phosphate-buffered saline injection, SI: *S. iniae* injection, VI: *V. harveyi* injection, SVI: *S. iniae* and *V. harveyi* injection, SIM: *S. iniae* immersion, VIM: *V. harveyi* immersion, SVIM: *S. iniae* and *V. harveyi* immersion

**Fig. 2:**
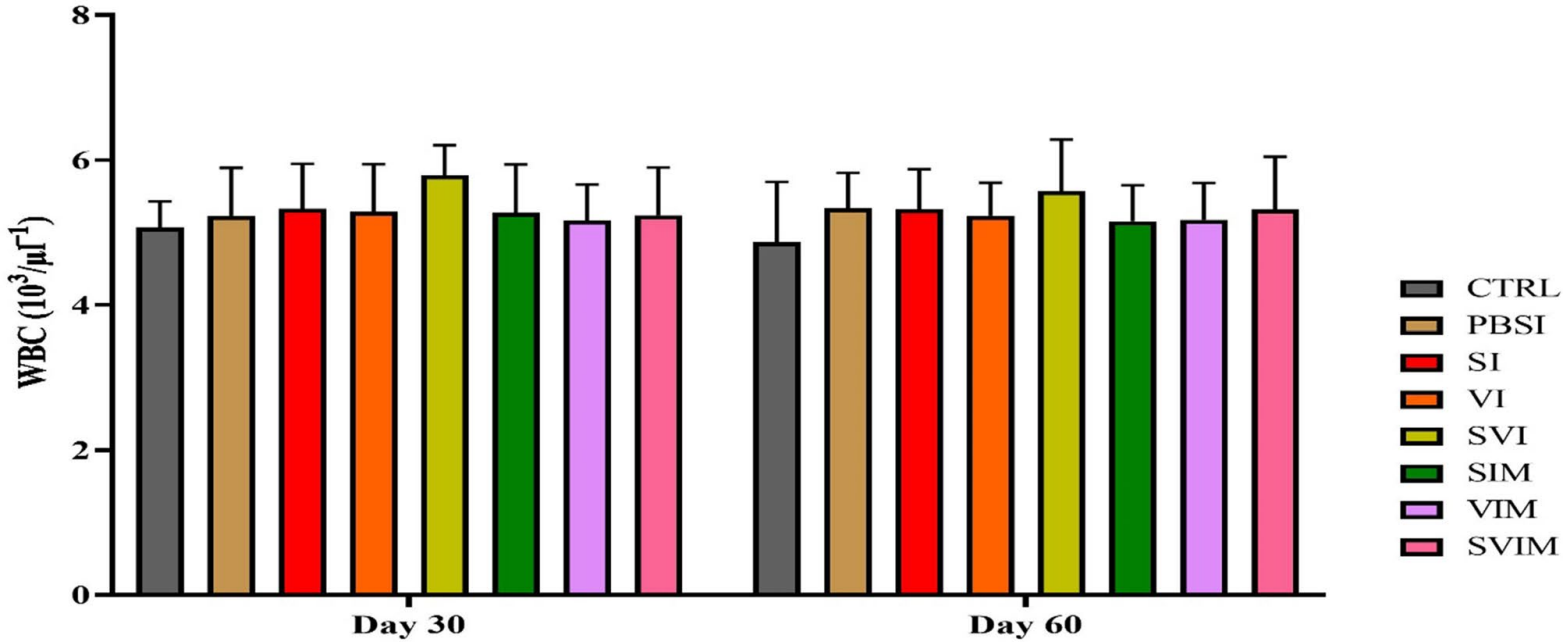
WBCs count in the groups vaccinated with *S. iniae* and *V. harveyi* in Asian seabass. The effect of time in all groups was not significant (*P* > 0.05). Interaction of time × treatment: *P* = 0.9967. Values are Mean ± SD (*n* = 9 fish per treatment). Statistical significance was measured using Tukey’s test and two-way ANOVA to show the difference groups over time. WBC: white blood cell, CTRL: control, PBSI: Phosphate-buffered saline injection, SI: *S. iniae* injection, VI: *V. harveyi* injection, SVI: *S. iniae* and *V. harveyi* injection, SIM: *S. iniae* immersion, VIM: *V. harveyi* immersion, SVIM: *S. iniae* and *V. harveyi* immersion

**Fig. 3:**
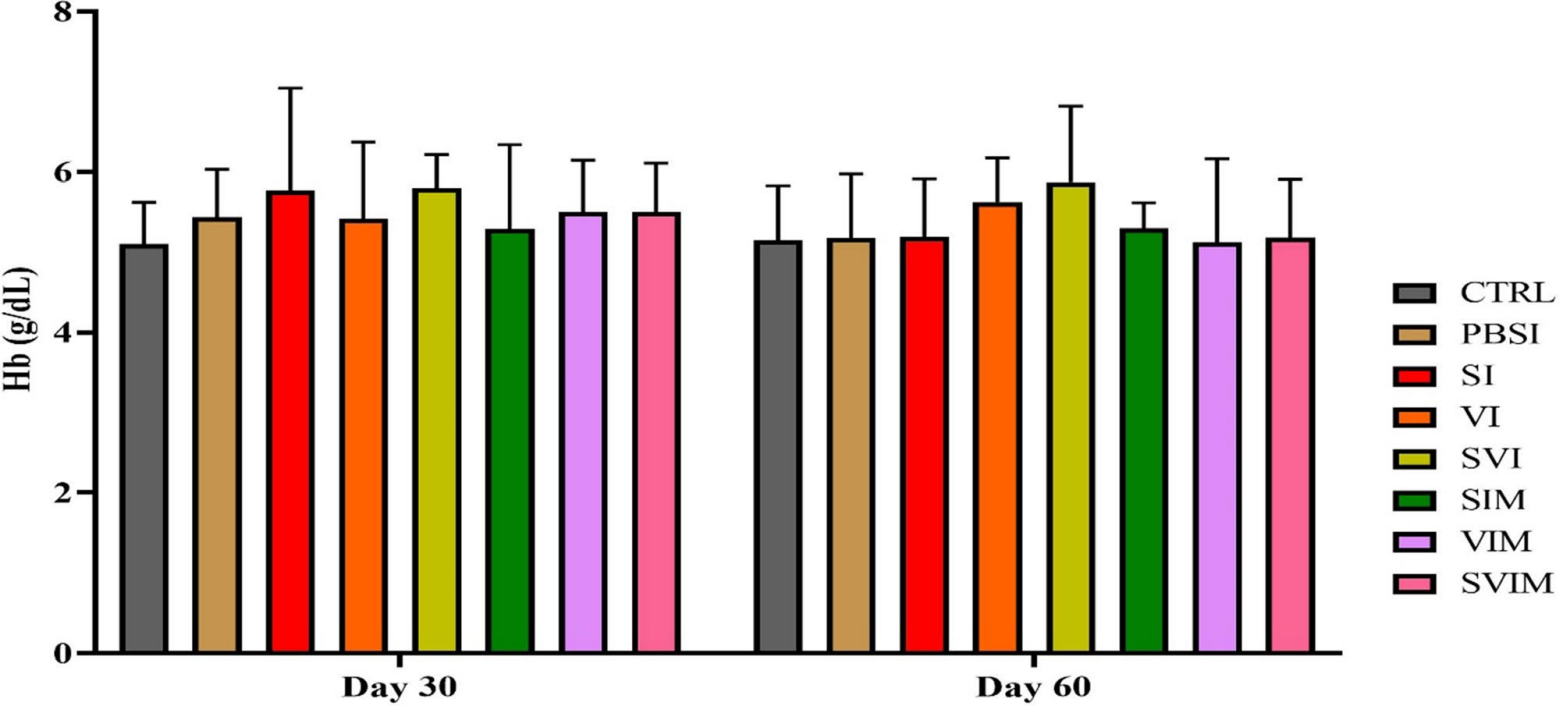
Hb concentration in the groups vaccinated with *S. iniae* and *V. harveyi* in Asian seabass. The effect of time in all groups was not significant (*P* > 0.05). Interaction of time × treatment: *P* = 0.9199. Values are Mean ± SD (*n* = 9 fish per treatment). Statistical significance was measured using Tukey’s test and two-way ANOVA to show the difference groups over time. Hb: hemoglobin, CTRL: control, PBSI: Phosphate-buffered saline injection, SI: *S. iniae* injection, VI: *V. harveyi* injection, SVI: *S. iniae* and *V. harveyi* injection, SIM: *S. iniae* immersion, VIM: *V. harveyi* immersion, SVIM: *S. iniae* and *V. harveyi* immersion

**Fig. 4:**
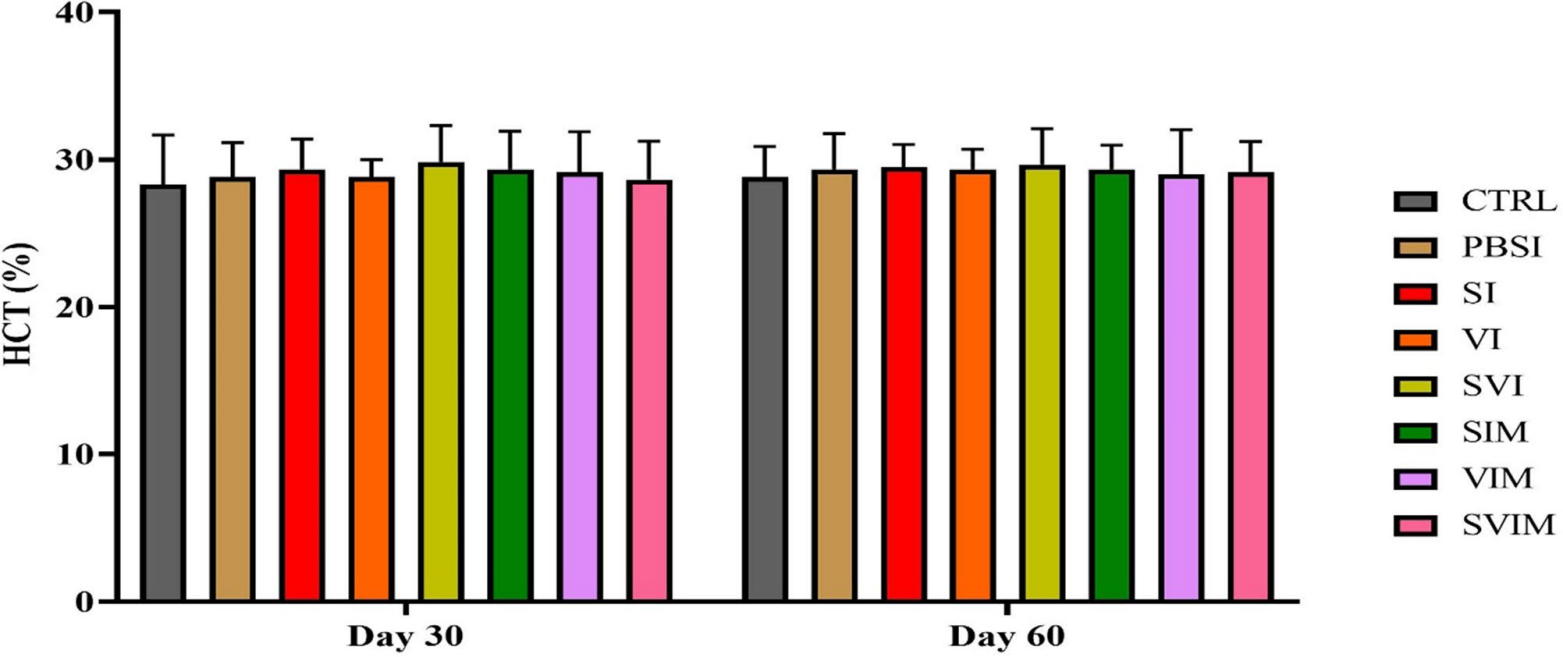
The percentage of Hct in the groups vaccinated with *V. harveyi* and *S. iniae* in Asian seabass. The effect of time in all groups was not significant (*P* > 0.05). Interaction of time × treatment: *P* = 0.9998. Values are Mean ± SD (*n* = 9 fish per treatment). Statistical significance was measured using Tukey’s test and two-way ANOVA to show the difference groups over time. Hct: hematocrit, CTRL: control, PBSI: Phosphate-buffered saline injection, SI: *S. iniae* injection, VI: *V. harveyi* injection, SVI: *S. iniae* and *V. harveyi* injection, SIM: *S. iniae* immersion, VIM: *V. harveyi* immersion, SVIM: *S. iniae* and *V. harveyi* immersion

**Fig. 5:**
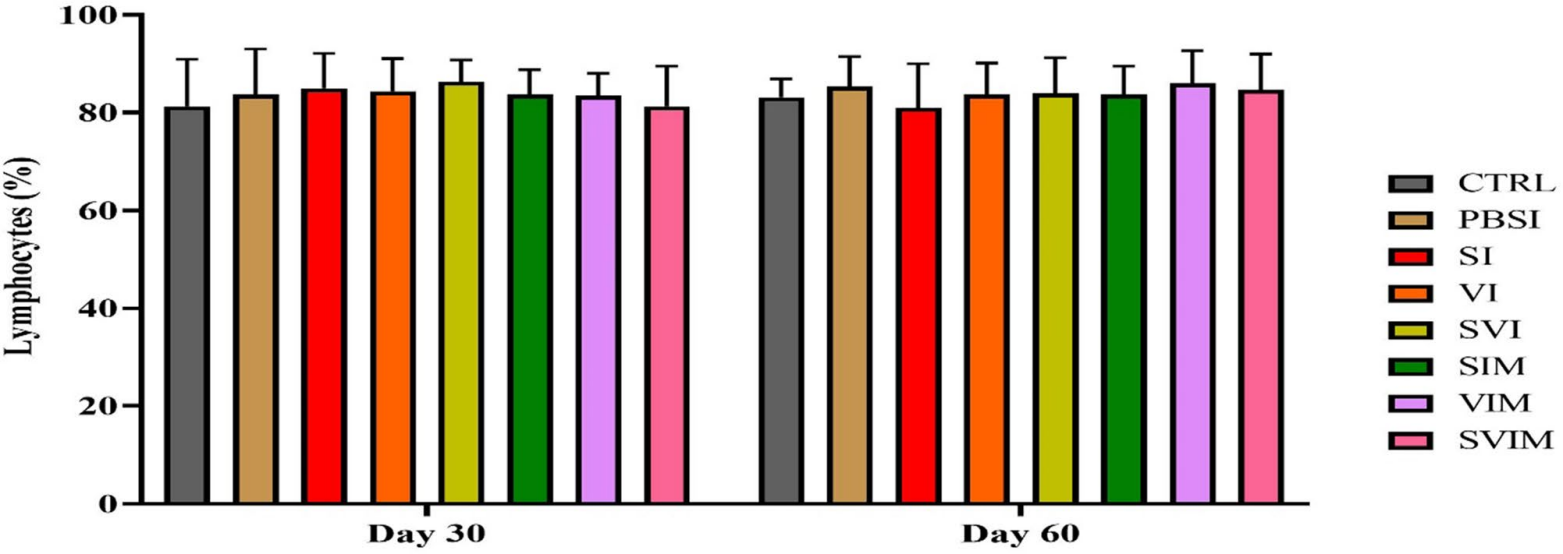
The percentage of Lymphocytes in the groups vaccinated with *S. iniae* and *V. harveyi* in Asian seabass. The effect of time in all groups was not significant (*P* > 0.05). Interaction of time × treatment: *P* = 0.9008. Values are Mean ± SD (*n* = 9 fish per treatment). Statistical significance was measured using Tukey’s test and two-way ANOVA to show the difference groups over time. Lym: lymphocytes, CTRL: control, PBSI: Phosphate-buffered saline injection, SI: *S. iniae* injection, VI: *V. harveyi* injection, SVI: *S. iniae* and *V. harveyi* injection, SIM: *S. iniae* immersion, VIM: *V. harveyi* immersion, SVIM: *S. iniae* and *V. harveyi* immersion

**Fig. 6:**
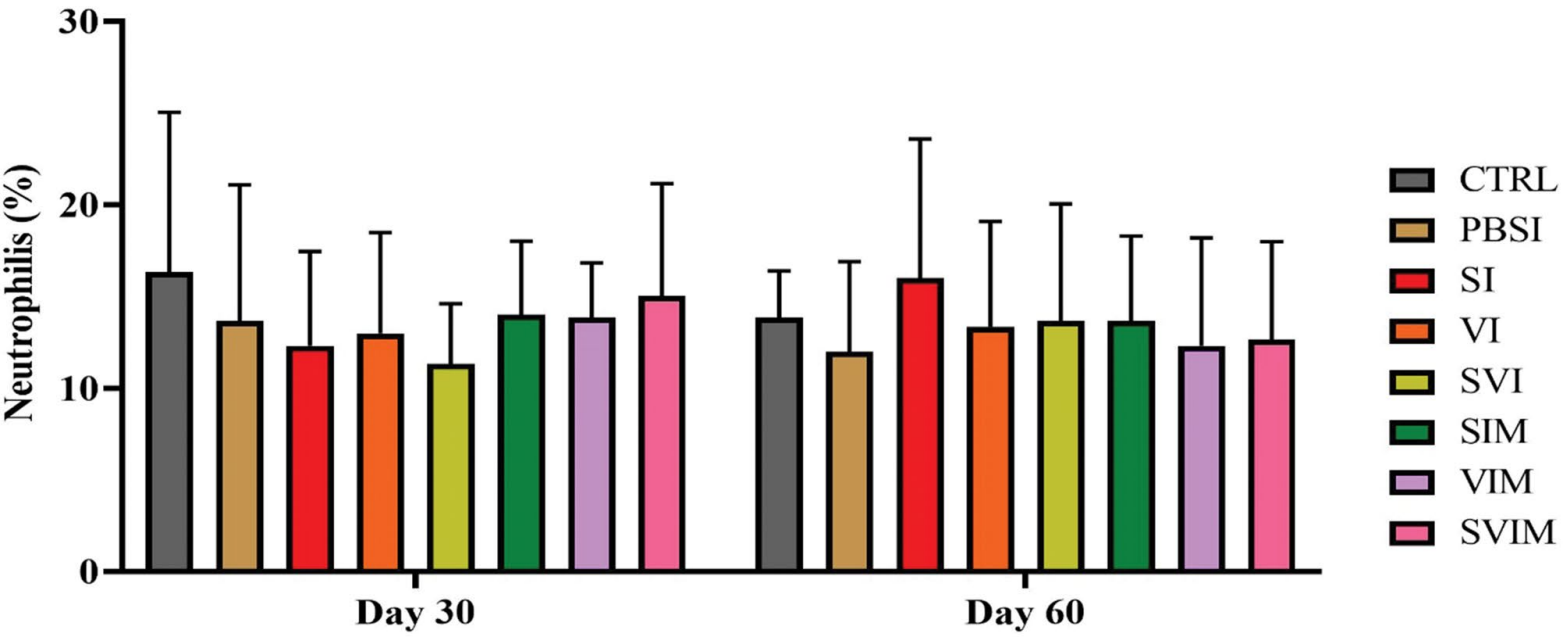
The percentage of Neutrophils in the groups vaccinated with *S. iniae* and *V. harveyi* in Asian seabass. The effect of time in all groups was not significant (*P* > 0.05). Interaction of time × treatment: *P* = 0.8478. Values are Mean ± SD (*n* = 9 fish per treatment). Statistical significance was measured using Tukey’s test and two-way ANOVA to show the difference groups over time. Neut: neutrophils, CTRL: control, PBSI: Phosphate-buffered saline injection, SI: *S. iniae* injection, VI: *V. harveyi* injection, SVI: *S. iniae* and *V. harveyi* injection, SIM: *S. iniae* immersion, VIM: *V. harveyi* immersion, SVIM: *S. iniae* and *V. harveyi* immersion

**Fig. 7:**
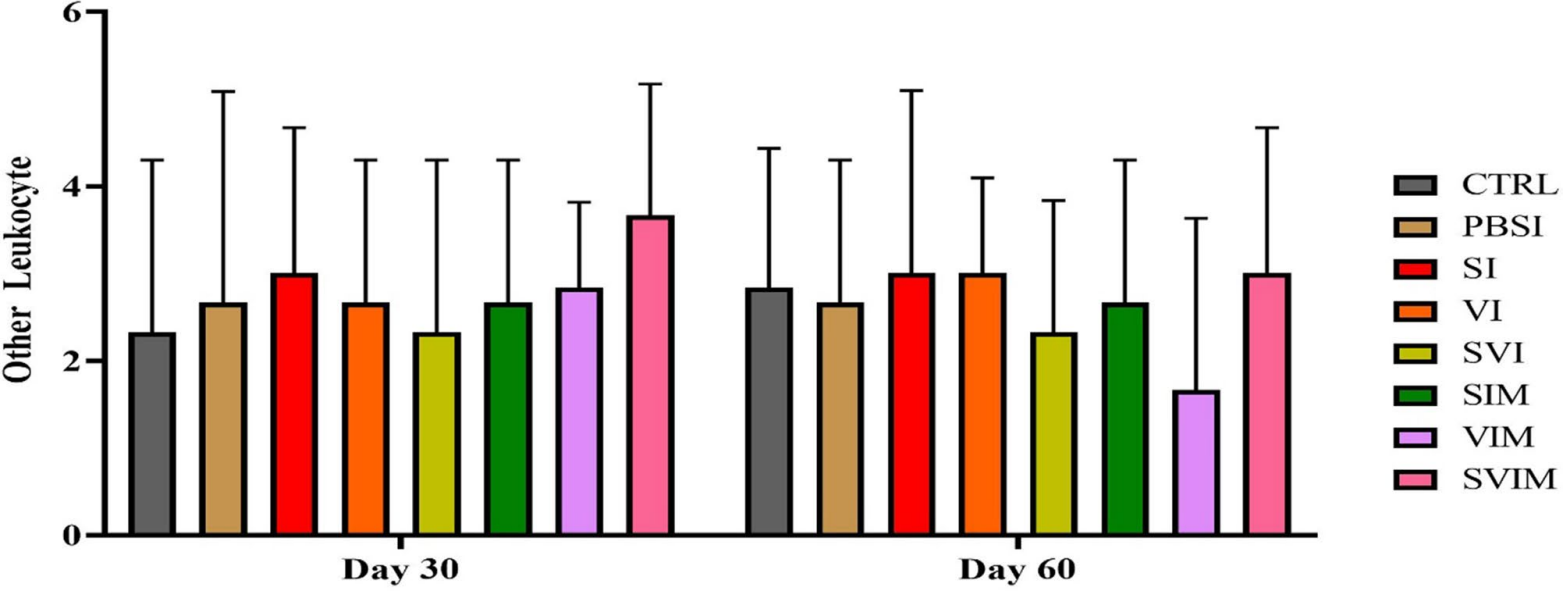
The other leukocytes in the groups vaccinated with *S. iniae* and *V. harveyi* in Asian seabass. The effect of time in all groups was not significant (*P* > 0.05). Interaction of time × treatment: *P* = 0.9535. Values are Mean ± SD (*n* = 9 fish per treatment). Statistical significance was measured using Tukey’s test and two-way ANOVA to show the difference groups over time. CTRL: control, PBSI: Phosphate-buffered saline injection, SI: *S. iniae* injection, VI: *V. harveyi* injection, SVI: *S. iniae* and *V. harveyi* injection, SIM: *S. iniae* immersion, VIM: *V. harveyi* immersion, SVIM: *S. iniae* and *V. harveyi* immersion

On the 30th day and the 60th day of sampling, there was no significant difference in blood parameters such as RBCs, WBCs, Hb, % Hct, Lym, Neut and other leukocytes in different groups (Figs. 1, 2, 3, 4, 5, 6 and 7, *P* > 0.05). Also, the main effect of time was not significant in all groups in the hematological factors (Figs. 1, 2, 3, 4, 5, 6 and 7, *P* > 0.05). The interaction between time and treatment RBC (*P* > 0.9909), WBC (*P* > 0.9967), Hb (*P* > 0.9199), %Hct (*P* > 0.9998), Lym (*P* > 0.9008), Neut (*P* > 0.8478) and other leukocytes (*P* > 0.9535) were not significant in any of the hematological parameters.

### Serum biochemical parameters

**Fig. 8:**
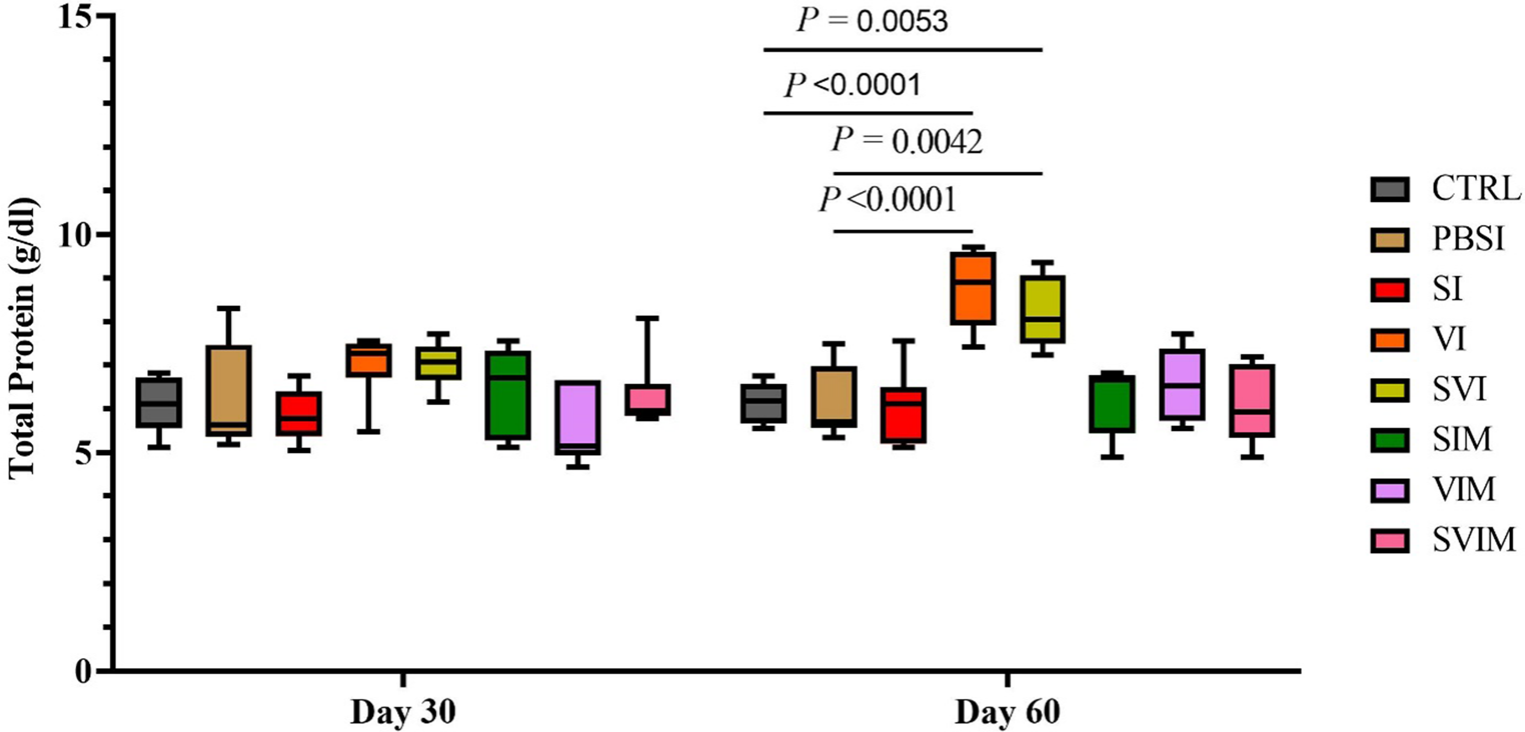
The activity of serum Total protein in the groups vaccinated with *S. iniae* and *V. harveyi* in Asian seabass. The effect of time in all groups was not significant (except VI group), (*P* > 0.05). Interaction of time × treatment: *P* = 0.0442. Values are Mean ± SD (*n* = 9 fish per treatment). Statistical significance was measured using Tukey’s test and two-way ANOVA to show the difference groups over time. CTRL: control, PBSI: Phosphate-buffered saline injection, SI: *S. iniae* injection, VI: *V. harveyi* injection, SVI: *S. iniae* and *V. harveyi* injection, SIM: *S. iniae* immersion, VIM: *V. harveyi* immersion, SVIM: *S. iniae* and *V. harveyi* immersion

**Fig. 9:**
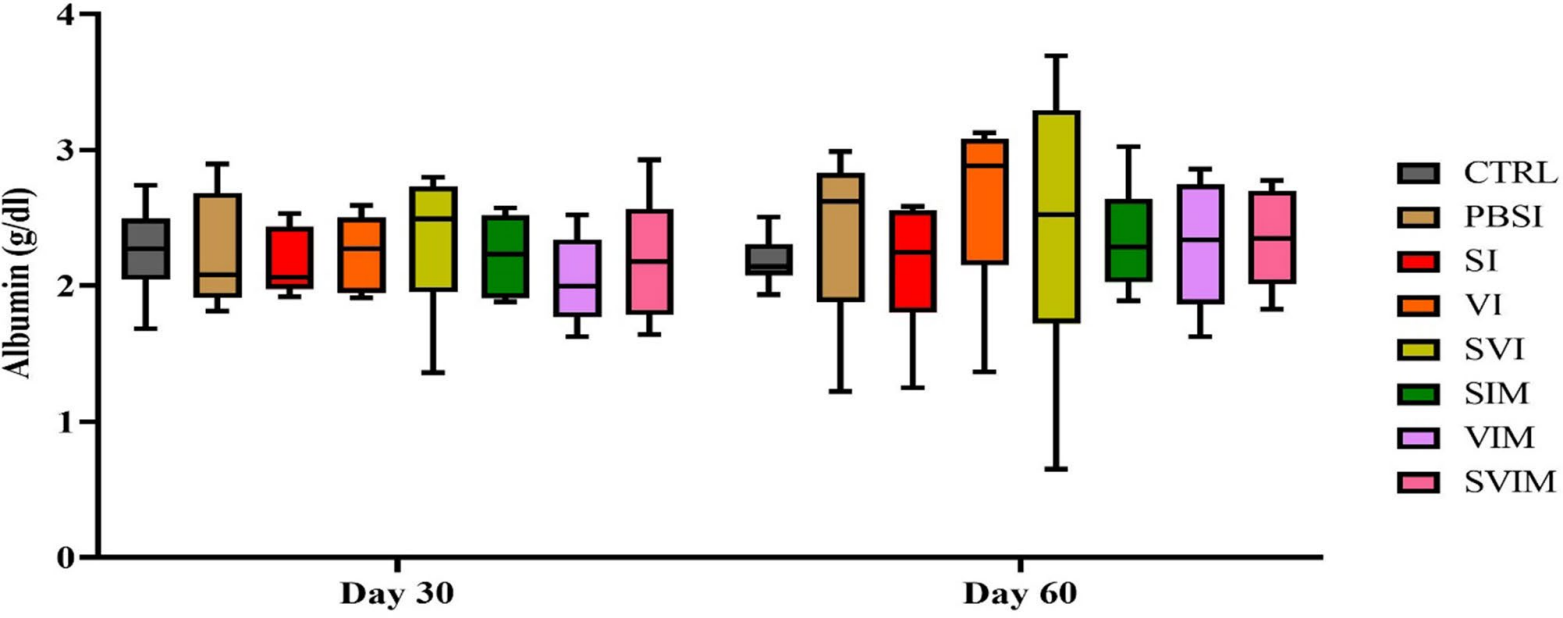
The activity of serum Albumin in the groups vaccinated with *S. iniae* and *V. harveyi* in Asian seabass. The effect of time in all groups was not significant (*P* > 0.05). Interaction of time × treatment: *P* = 0.9711. Values are Mean ± SD (*n* = 9 fish per treatment). Statistical significance was measured using Tukey’s test and two-way ANOVA to show the difference groups over time. CTRL: control, PBSI: Phosphate-buffered saline injection, SI: *S. iniae* injection, VI: *V. harveyi* injection, SVI: *S. iniae* and *V. harveyi* injection, SIM: *S. iniae* immersion, VIM: *V. harveyi* immersion, SVIM: *S. iniae* and *V. harveyi* immersion

**Fig. 10:**
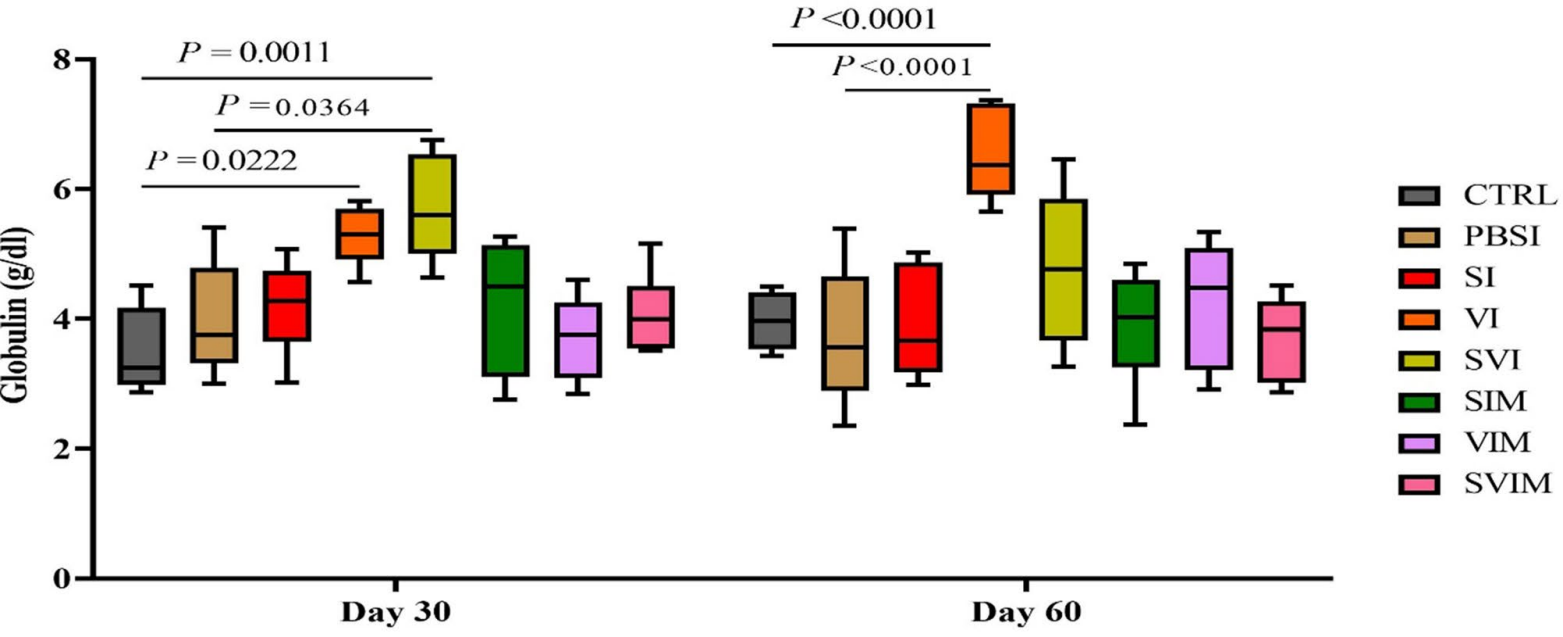
The activity of serum Globulin in the groups vaccinated with *S. iniae* and *V. harveyi* in Asian seabass. The effect of time in all groups was not significant (*P* > 0.05). Interaction of time × treatment: *P* = 0.0508. Values are Mean ± SD (*n* = 9 fish per treatment). Statistical significance was measured using Tukey’s test and two-way ANOVA to show the difference groups over time. CTRL: control, PBSI: Phosphate-buffered saline injection, SI: *S. iniae* injection, VI: *V. harveyi* injection, SVI: *S. iniae* and *V. harveyi* injection, SIM: *S. iniae* immersion, VIM: *V. harveyi* immersion, SVIM: *S. iniae* and *V. harveyi* immersion

On the 30th day, there was no significant difference in the amount of Tp in all groups compared to the CTRL group, but on the 60th day, there was a significant difference in the amount of Tp in VI and SVI compared to the CTRL and PBSI groups (Fig. 8, *P* < 0.05). The main effect of time in all groups (except the VI group) was not significant in the activity of serum Tp (Fig. 8, *P* > 0.05), but the interaction between treatment and time was significant in the activity of serum Tp (*P* < 0.0288). On the 30th and 60th days, there was no significant difference in serum Alb activity in all groups compared to the CTRL group (Fig. 9, *P* > 0.05). The main effect of time in all groups (Fig. 10, *P* > 0.05), as well as the interaction between treatment and time, had no significant difference in activity of serum Alb (*P* > 0.9711). The activity of serum Glb on the 30th day (VI and SVI groups compared to the CTRL group and SVI compared to the PBSI group) and the 60th day (VI group compared to the CTRL and PBSI groups) had a significant increase (Fig. 10, *P* < 0.05). The main effect of time in all groups (Fig. 9, *P* > 0.05) and the interaction between treatment and time in serum Glb activity were not significant (*P* > 0.0508).

### Non-specific immune parameters

**Fig. 11:**
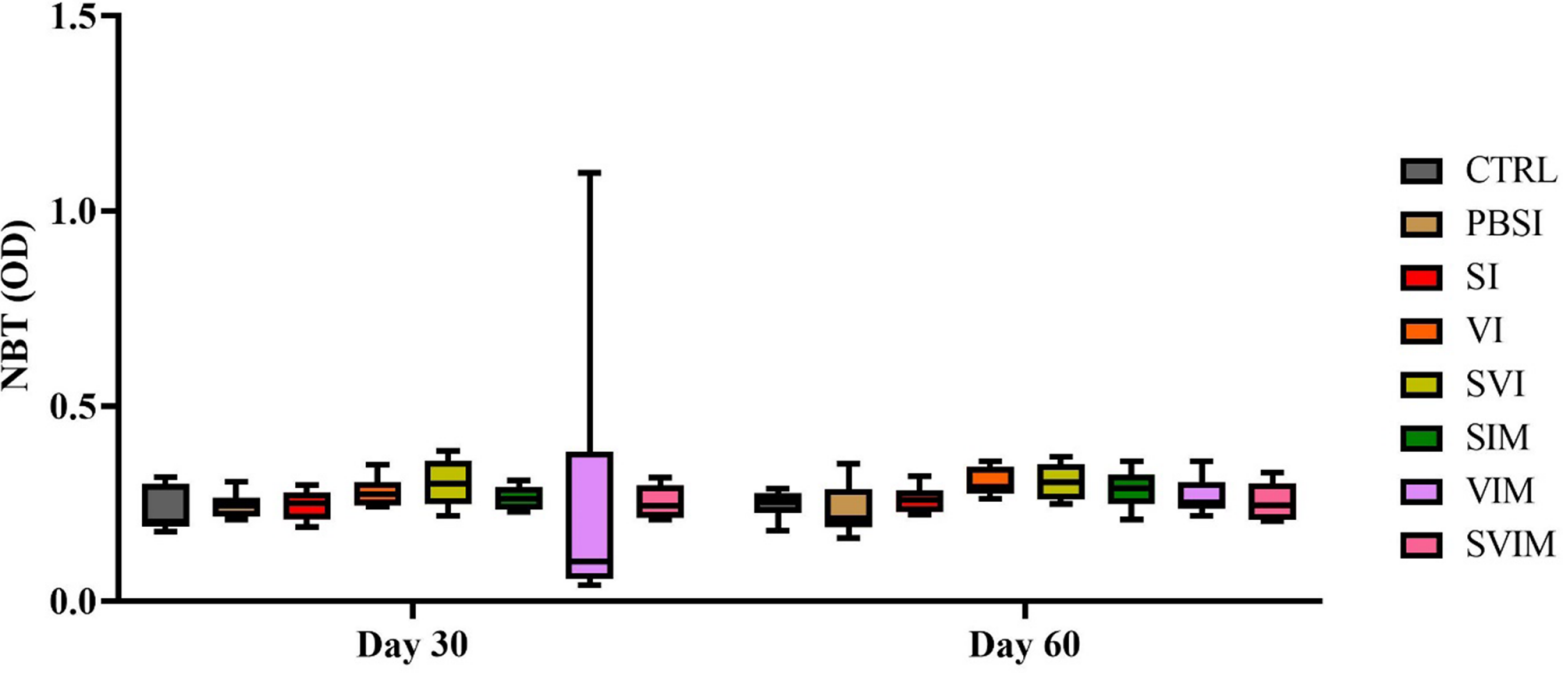
The activity of serum NBT in the groups vaccinated with *S. iniae* and *V. harveyi* in Asian seabass. The effect of time in all groups was not significant (*P* > 0.05). Interaction of time × treatment: *P* > 0.9999. Values are Mean ± SD (*n* = 9 fish per treatment). Statistical significance was measured using Tukey’s test and two-way ANOVA to show the difference groups over time. NBT: nitroblue tetrazolium, CTRL: control, PBSI: Phosphate-buffered saline injection, SI: *S. iniae* injection, VI: *V. harveyi* injection, SVI: *S. iniae* and *V. harveyi* injection, SIM: *S. iniae* immersion, VIM: *V. harveyi* immersion, SVIM: *S. iniae* and *V. harveyi* immersion

**Fig. 12:**
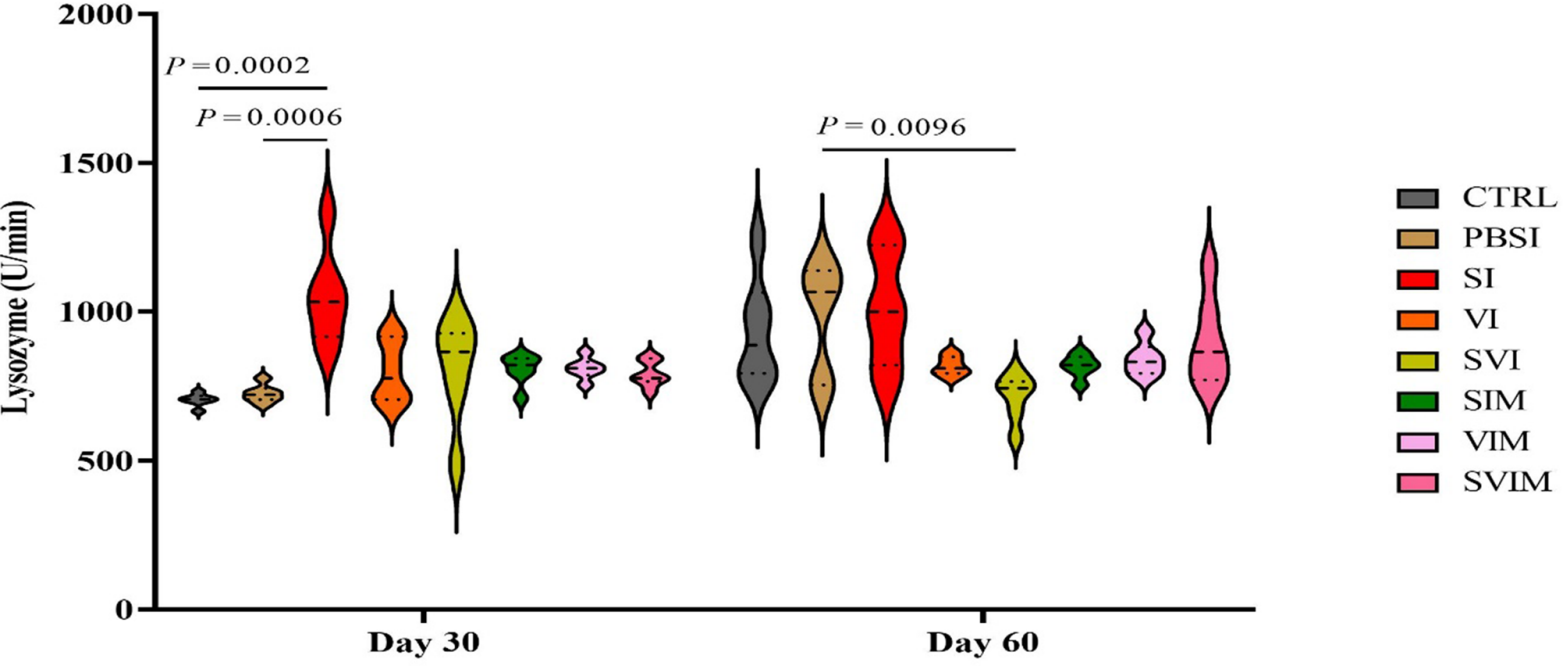
The activity of serum lysozyme in the groups vaccinated with *S. iniae* and *V. harveyi* in Asian seabass. The effect of time in all groups was not significant (*P* > 0.05). Interaction of time × treatment: *P* = 0.0025. Values are Mean ± SD (*n* = 9 fish per treatment). Statistical significance was measured using Tukey’s test and two-way ANOVA to show the difference groups over time. CTRL: control, PBSI: Phosphate-buffered saline injection, SI: *S. iniae* injection, VI: *V. harveyi* injection, SVI: *S. iniae* and *V. harveyi* injection, SIM: *S. iniae* immersion, VIM: *V. harveyi* immersion, SVIM: *S. iniae* and *V. harveyi* immersion

**Fig. 13:**
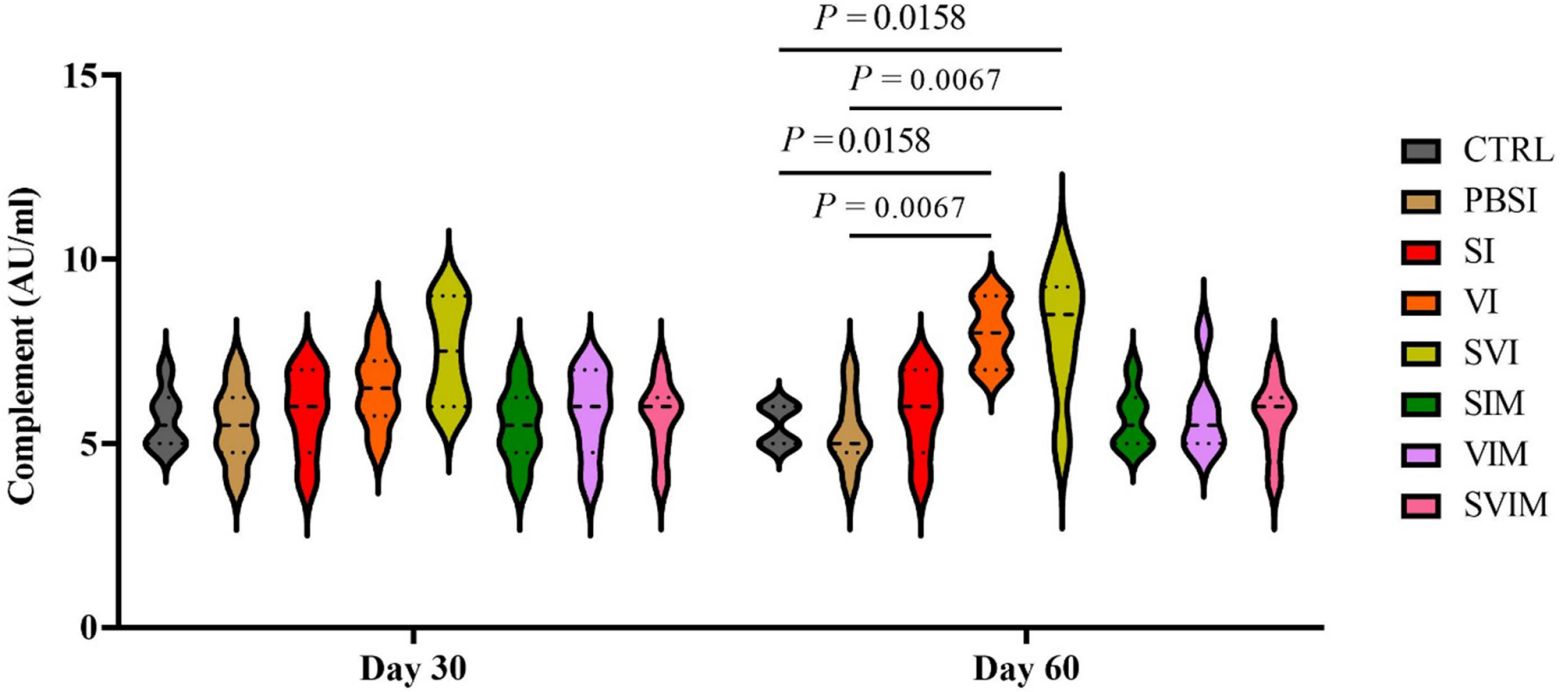
The activity of serum complement components in the groups vaccinated with *S. iniae* and *V. harveyi* in Asian seabass. The effect of time in all groups was not significant (*P* > 0.05). Interaction of time × treatment: *P* = 0.6217. Values are Mean ± SD (*n* = 9 fish per treatment). Statistical significance was measured using Tukey’s test and two-way ANOVA to show the difference groups over time. CTRL: control, PBSI: Phosphate-buffered saline injection, SI: *S. iniae* injection, VI: *V. harveyi* injection, SVI: *S. iniae* and *V. harveyi* injection, SIM: *S. iniae* immersion, VIM: *V. harveyi* immersion, SVIM: *S. iniae* and *V. harveyi* immersion

No significant difference was observed in the activity of serum NBT on the 30th and 60th days of sampling in all groups compared to the CTRL group (Fig. 11, *P* > 0.05). The main impact of time in all groups (Fig. 11, *P* > 0.05), as well as the interaction between treatment and time, had no significant difference in serum NBT (*P* > 0.9999). The activity of serum lysozyme was significantly increased on the 30th day (SI group compared to the CTRL and PBSI group), (Fig. 12, *P* < 0.05) and on the 60th day of sampling (SVI group compared to the CTRL group), (Fig. 12, *P* < 0.05). The main impact of time in all groups (except PBSI) was not significant in the activity of serum lysozyme (Fig. 12, *P* > 0.05). The interaction between treatment and time was significant in the activity of serum lysozyme (*P* < 0.0025). On the 30th day of sampling, no significant difference was observed in the amount of serum complement in all groups compared to the CTRL group (Fig. 13, *P* > 0.05), but on the 60th day of sampling, a significant increase in serum complement levels was observed in the VI and SVI groups compared to the CTRL and PBSI groups (Fig. 13, *P* < 0.05). The main impact of time was not significant in all groups compared to the CTRL group in activity of serum complement (Fig. 13, *P* > 0.05). Also, no significant difference was observed in the interaction between treatment and time in activity of serum complement (*P* > 0.6217).

### Serum bactericidal activity

**Fig. 14:**
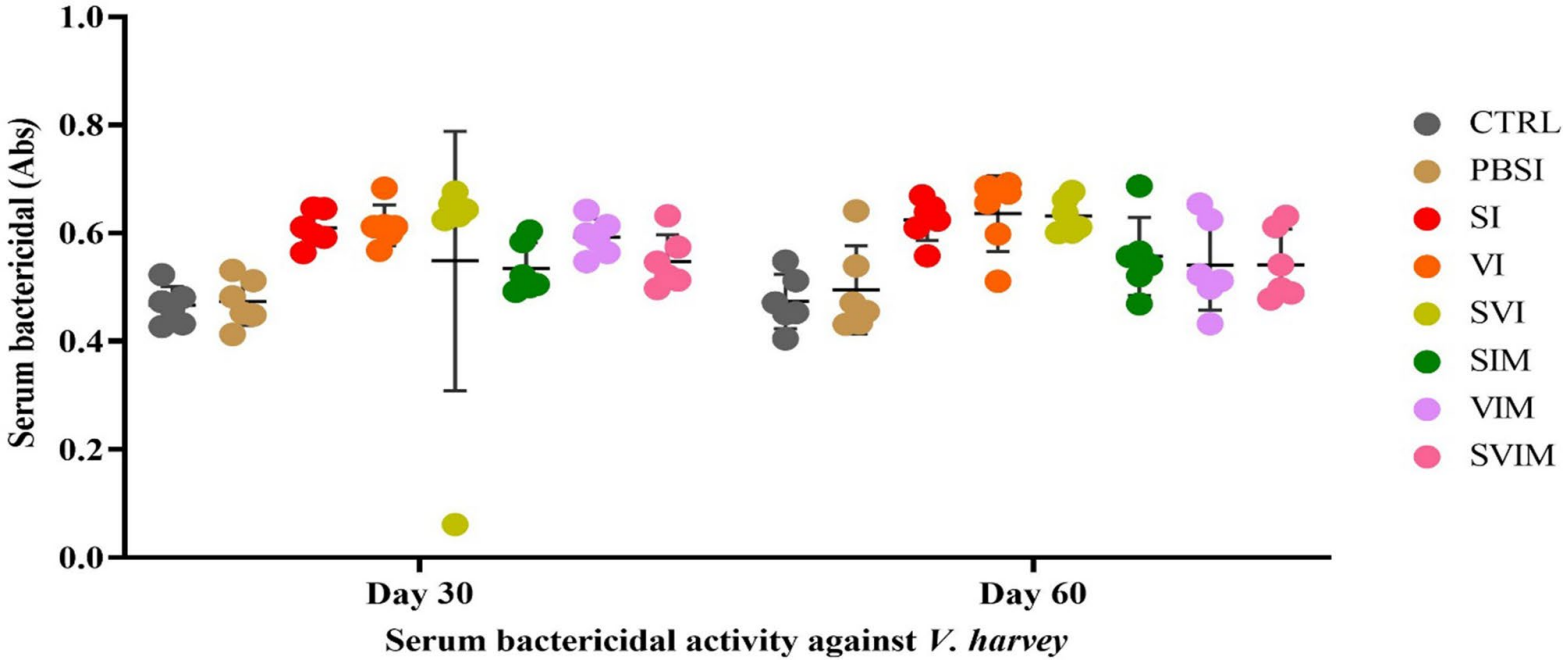
Serum bactericidal activity against *V. harveyi* in the groups vaccinated with *S. iniae* and *V. harveyi* in Asian seabass. The effect of time in all groups was not significant (*P* > 0.05). Interaction of time × treatment: *P* = 0.7128. Values are Mean ± SD (*n* = 9 fish per treatment). Statistical significance was measured using Tukey’s test and two-way ANOVA to show the difference groups over time. CTRL: control, PBSI: Phosphate-buffered saline injection, SI: *S. iniae* injection, VI: *V. harveyi* injection, SVI: *S. iniae* and *V. harveyi* injection, SIM: *S. iniae* immersion, VIM: *V. harveyi* immersion, SVIM: *S. iniae* and *V. harveyi* immersion

The bactericidal activity of serum against *V. harveyi* on the 30th and 60th days of sampling in all groups was not significant compared to the CTRL group (Fig. 14, *P* > 0.05). The main impact of time (Fig. 14, *P* > 0.05), and the interaction of time in the treatment were not significant in the bactericidal activity of serum against *V. harveyi* (*P* > 0.7128).

### Antibody titer

**Fig. 15:**
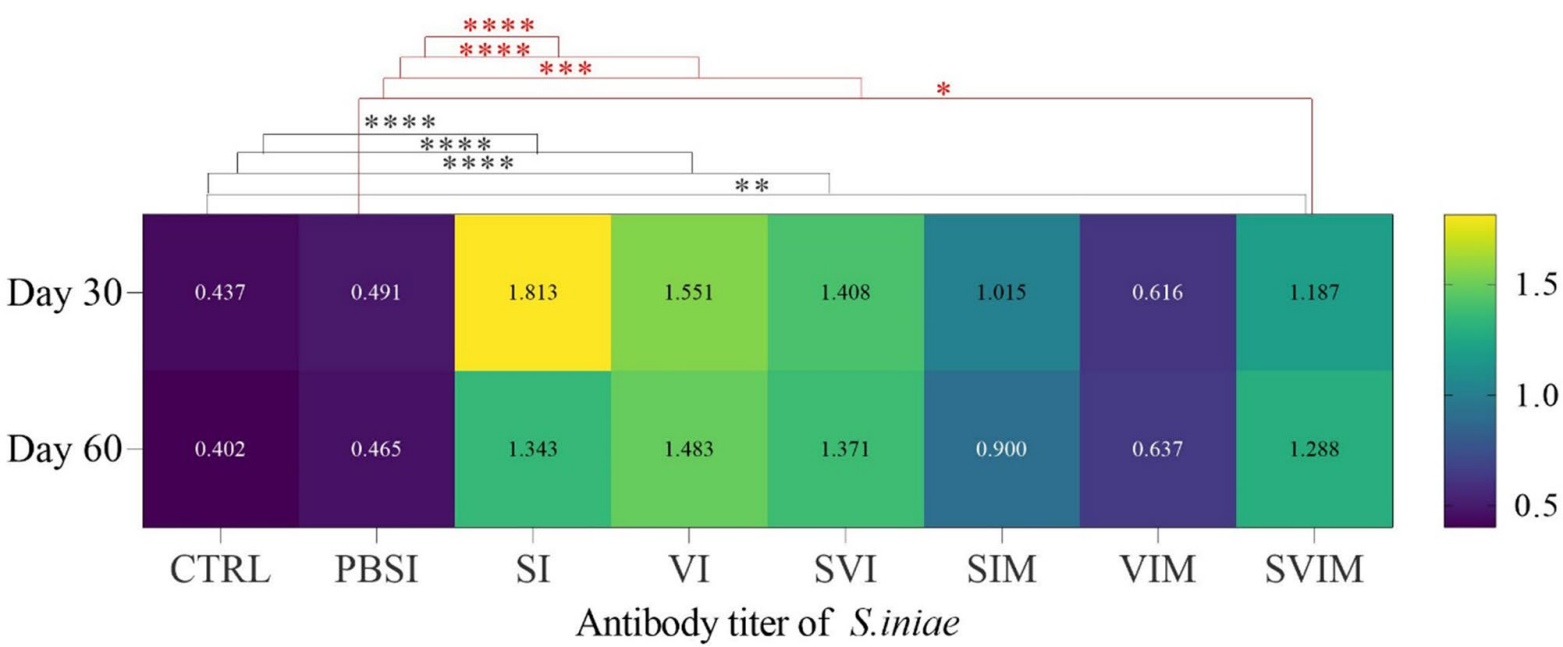
Antibody titer against *S. iniae* in the groups vaccinated with *S. iniae* and *V. harveyi* in Asian seabass. The effect of time in all groups was not significant (*P* > 0.05). Interaction of time × treatment: *P* = 0.4945. Values are Mean ± SD (*n* = 9 fish per treatment). Statistical significance was measured using Tukey’s test and two-way ANOVA to show the difference groups over time. CTRL: control, PBSI: Phosphate-buffered saline injection, SI: *S. iniae* injection, VI: *V. harveyi* injection, SVI: *S. iniae* and *V. harveyi* injection, SIM: *S. iniae* immersion, VIM: *V. harveyi* immersion, SVIM: *S. iniae* and *V. harveyi* immersion. *: *P* = 0.0164, **: *P* = 0.0060, ***: *P* = 0.0002. ****: *P* < 0.0001

**Fig. 16:**
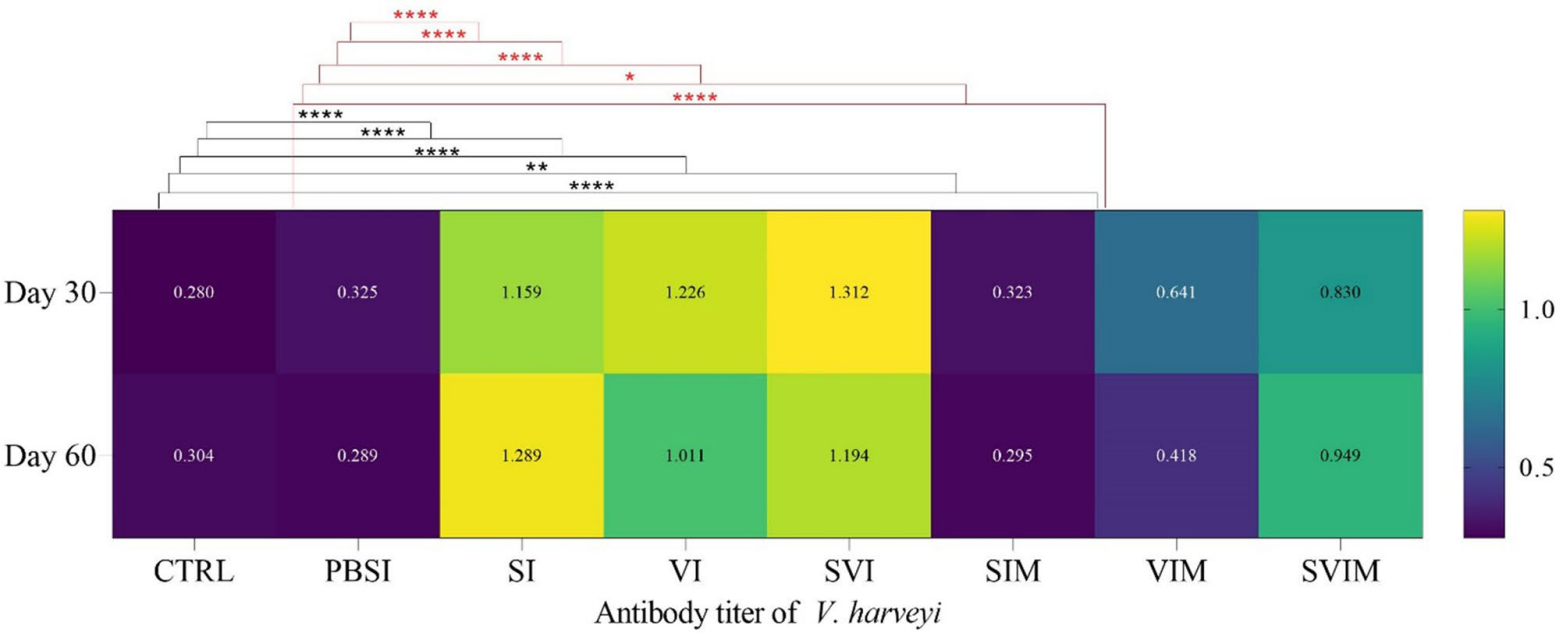
Antiboby titer against *V. harveyi* in the groups vaccinated with *S. iniae* and *V. harveyi* in Asian seabass. The effect of time in all groups was not significant (*P* > 0.05). Interaction of time × treatment: *P* = 0.0186. Values are Mean ± SD (*n* = 9 fish per treatment). Statistical significance was measured using Tukey’s test and two-way ANOVA to show the difference groups over time. CTRL: control, PBSI: Phosphate-buffered saline injection, SI: *S. iniae* injection, VI: *V. harveyi* injection, SVI: *S. iniae* and *V. harveyi* injection, SIM: *S. iniae* immersion, VIM: *V. harveyi* immersion, SVIM: *S. iniae* and *V. harveyi* immersion. *: *P* = 0.0263, **: *P* = 0.0047, ****: *P* < 0.0001

The serum antibody titer against *S. iniae* on the 30th and 60th days of sampling had a significant difference in the SI, VI, SVI and SVIM groups compared to the CTRL and PBSI groups (Fig. 15, *P* < 0.05). The main effect of time on the serum antibody titer against *S. iniae* in all groups was not significant on day 30 compared to day 60 (Fig. 15, *P* > 0.05). Also, the serum antibody titer against *S. iniae* was not significant in the interaction between treatment and time (*P* = 0.4945). The serum antibody titer against *V. harveyi* on the 30th day (in the SI, VI, SVI, VIM and SVIM groups) and on the 60th day (in the SI, VI, SVI and SVIM groups) had a significant increase compared to the CTRL and PBSI groups (Fig. 16, *P* < 0.05). The main effect of time in serum antibody titer against *V. harveyi* was not significant in all groups on day 30 compared to day 60 (Fig. 16, *P* < 0.05), but serum antibody titer against *V. harveyi* was significant in the interaction between treatment and time (*P* = 0.0186).

### Survival post-challenge

**Fig. 17:**
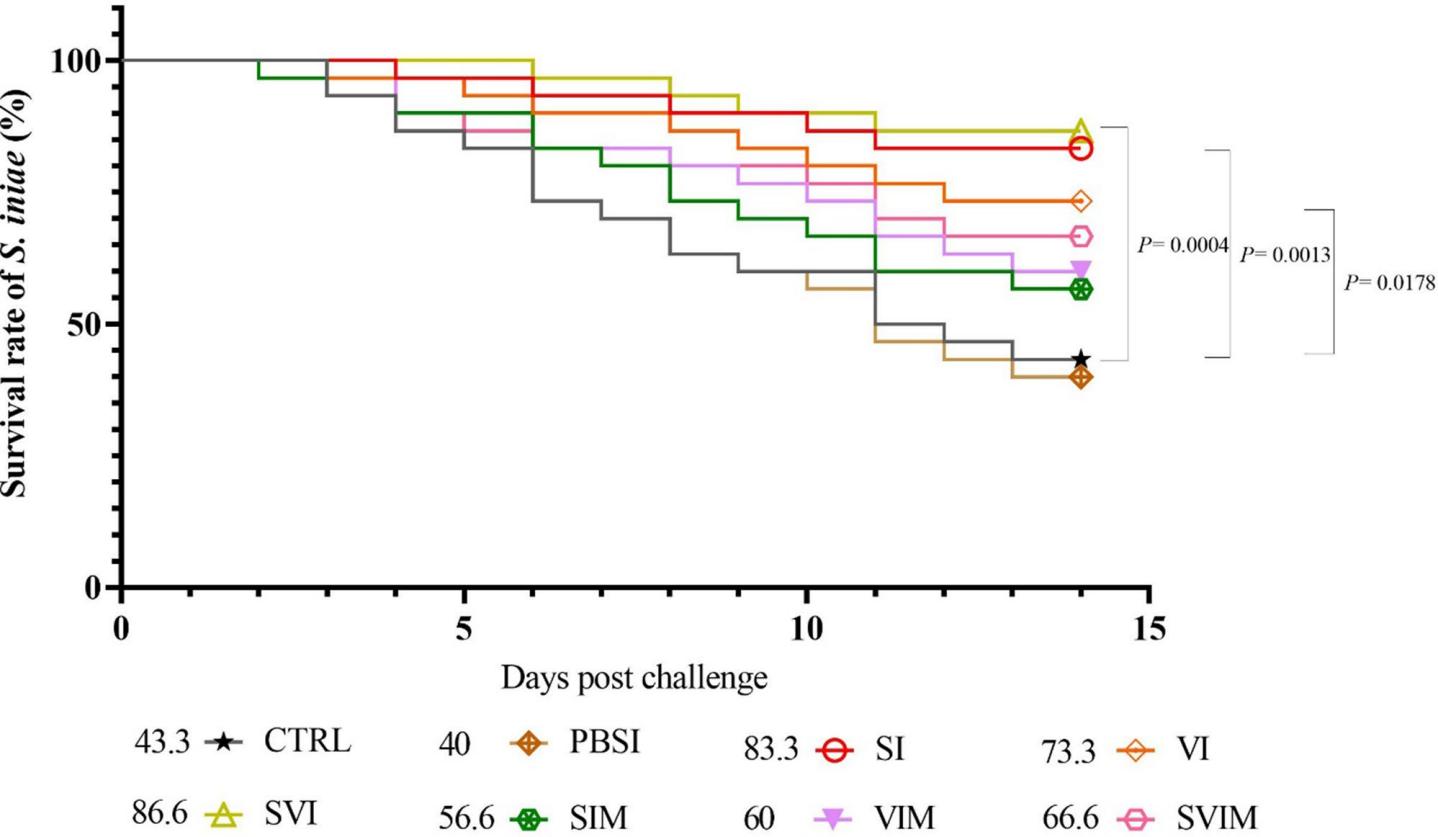
Cumulative survival rate of Asian seabass after 14 days of challenge with *S. iniae* (Kaplan-Meier curve). Values are Mean ± SD (*n* = 30 fish per treatment). Statistical significance was measured using Tukey’s test and one-way ANOVA to show the different groups over time. *S. iniae*: *Streptococcus iniae*, CTRL: control, PBSI: Phosphate-buffered saline injection, SI: *S. iniae* injection, VI: *V. harveyi* injection, SVI: *S. iniae* and *V. harveyi* injection, SIM: *S. iniae* immersion, VIM: *V. harveyi* immersion, SVIM: *S. iniae* and *V. harveyi* immersion

**Fig. 18:**
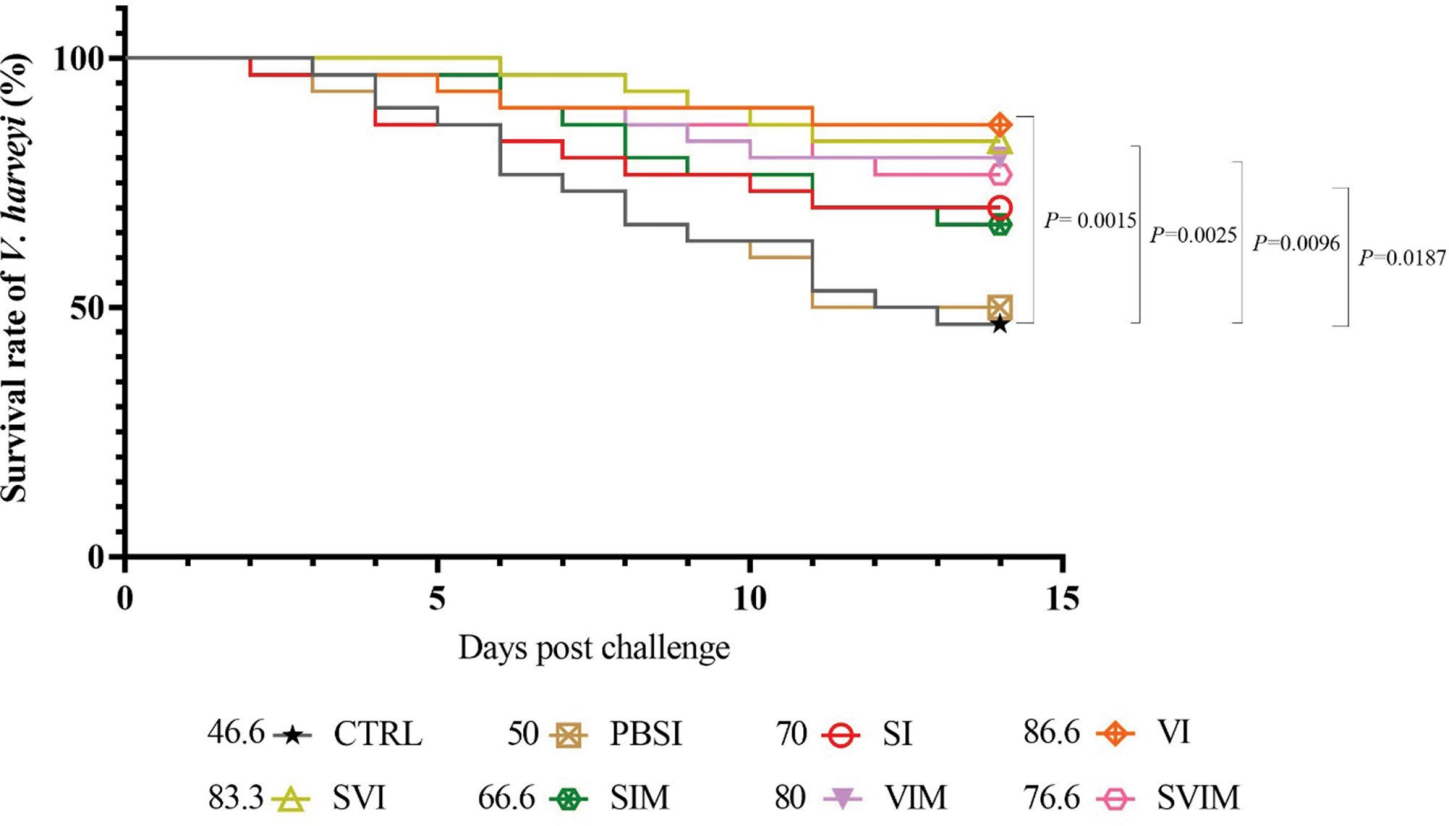
Cumulative survival rate of Asian seabass after 14 days of challenge with *V. harveyi* (Kaplan-Meier curve). Values are Mean ± SD (*n* = 30 fish per treatment). Statistical significance was measured using Tukey’s test and one-way ANOVA to show the different groups over time. *V. harveyi*: *Vibrio harveyi*, CTRL: control, PBSI: Phosphate-buffered saline injection, SI: *S. iniae* injection, VI: *V. harveyi* injection, SVI: *S. iniae* and *V. harveyi* injection, SIM: *S. iniae* immersion, VIM: *V. harveyi* immersion, SVIM: *S. iniae* and *V. harveyi* immersion

After 14 days of challenge with *S. iniae* bacteria, SI (*P* = 0.0013), VI (*P* = 0.0178) and SVI (*P* = 0.0004) groups had a significant increase in relative percentage survival (RPS) compared to the CTRL and PBSI groups (Fig. 17). The SVI group with 86.6% highest survival rate and PBSI group with 40% lowest survival rate (SR) are shown in Fig. 17. Also, after 14 days of challenge with *V. harveyi*, VI (*P* = 0.0015), SVI (*P* = 0.0025), VIM (*P* = 0.0096) and SVIM (*P* = 0.0187) groups had a significant increase in RPS compared to the CTRL and PBSI groups (Fig. 18). The VI group with 86.6% highest SR and CTRL group with 46.6% lowest SR are shown in Fig. 18.

## Discussion

Many vaccines have been developed recently to protect fish in aquaculture from acute bacterial infections such as Vibriosis [14], Streptococcosis [20], Edwardsiellosis [21], Yersiniosis [6] and Furunculosis [22]. The bivalent and polyvalent vaccines are safe for fish and consumers, have long-term immunity, easy to use, cost-effective, and are applicable for co-infections [6, 14]. Therefore, in the present study, we used a bivalent formalin-inactivated vaccine to protect against Streptococcosis and Vibriosis by injection and immersion methods, which might cover all the requirements of a potential vaccine. Our study showed that the bivalent vaccine does not have a significant effect on blood parameters on the 30th and 60th day. The blood parameters are considered as a vital indicator of health status in aquatic animals. In line with our results, the effect of *Aeromonas salmonicida* vaccine in pikeperch (*Sander lucioperca*) was investigated in a study [23]. The results showed that this vaccine had no significant effect on blood parameters such as WBC, RBC, %HCT, MCH and MCHC [23]. In another study, the effect of two different adjuvants on *Streptococcus agalactia*e vaccine was investigated in Nile tilapia. The results showed that this vaccine has no significant effect on blood parameters such as %HCT, MCV, MCHC, MCH and total erythrocytes [24].

Serum biochemical parameters such as Tp, Alb and Glb are considered as one of the effective indicators of humoral immunity, pathological and welfare status of fish [25]. In the present study, it has been shown that Tp in the vaccinated groups (VI and SVI on the 60th day) and Glb (in the VI and SVI groups on the 30th day and VI on the 60th day) were significant increase compared to the control group. Consistent with our results, the increase of mucosal immune response in *Labeo rohita* was investigated using recombinant bicistronic nano DNA vaccine priming against *Edwarsiella tarda*. The results showed that the recombinant bicistronic nano DNA vaccine priming against *E. tarda* has a significant difference in blood biochemical factors such as Tp, Alb and Glb of blood serum [26]. In another study, the effect of Streptococcosis vaccine in Nile tilapia had a significant increase in Glb and Tp, but did not affect Alb [24]. Globulin is necessary to regulate and increase the efficiency of the fish’s immune system, and an increase in the amount of globulin indicates a strong innate immune system [27].

One of the important defense mechanisms against pathogenic bacteria is phagocytic activity by phagocytic cells. During the respiratory burst activity, phagocytic cells can produce superoxide anions, which are toxic forms of oxygen and lead to the elimination of bacteria [28]. In this study, vaccinated fish did not show any significant difference in blood NBT levels. Contrary to our results, the increase in blood NBT levels in the inactivated vaccine encapsulated with nano-chitosan against *A. hydrophila* in rainbow trout (*Oncorhynchus mykiss*) [29], oral polylactic-co-glycolic acid nano-encapsulated DNA vaccine against *A. hydrophila* in common carp [30] and Vibriosis vaccine in turbot *Scophthalmus maximus* [31] have been reported. The NBT index is one of the non-specific immune response indices to evaluate the level of phagocytosis. In the final stage of phagocytosis or intracellular killing, the creation of respiratory burst activity, which is under the influence of free radicals produced in phagocytic cells, is performed by nitrobutrazolium. Since vaccination with killed bacteria has the greatest effect on humoral immunity. It is possible that some non-specific immunity indicators are significantly affected by vaccination. On the other hand, many other environmental and physiological factors have an effect on the amount of phagocytosis (apart from the prescribed vaccine) and the increase in NBT activity is actually one of the factors affecting phagocytosis, which in the current research, despite a mild increase, was not statistically significant.

The complement system can cause activation, phagocytosis, chemotaxis, inflammatory response, opsonization and immunocytes for pathogens and plays a major role in the innate immunity of fish [32]. In the present study, serum complement activity on the 60th day of sampling was significantly increased in the VI and SVI vaccinated groups compared to the CTRL and PBSI groups. Consistent with our results, the *V. mimicus* vaccine in *Carassius auratus* [33], polyvalent streptococcosis/yersiniosis vaccine in rainbow trout [6] and bivalent *V. scophthalmi* and *A. salmonicida* vaccines in turbot (*S. maximus*) [34] had a positive and significant effect on the complement system of fish. The increase of complement activity after immunization with both monovalent and bivalent vaccines, proved the effectiveness of inactivated vaccines against *S. iniae* and *V. harveyi* in Asian seabass.

Non-specific immune response is the first defense line in fish, which plays an important role in the secondary or specific immune system and the regulation of body homeostasis [35]. Due to its close relationship with leukocytes, lysozyme is an accepted and preferred marker of immune response, which is produced by macrophages and many other immune stimuli in response to pathogenic components [36]. In the current study, serum lysozyme activity increased significantly on day 30 (SI group compared to CTRL and PBSI group) and on day 60 of sampling (SVI group compared to CTRL group). In line with our results, in a study, the effect of *V. harveyi* vaccine combined with chitosan and astragalus polysaccharide in ♀*Epinephelus fuscoguttatus* × ♂*Epinephelus lanceolatus* had a significant effect on lysozyme activity compared to the control group [37]. Several studies prove the effect of polyvalent vaccine on increasing lysozyme activity, for example, the combination of *V. anguillarum* and *Edwardsiella piscicida* vaccine in turbot [38], the polyvalent yersiniosis and streptococcosis/lactococcosis vaccine in *O. mykiss* [6] and the bivalent *A. salmonicida* and *E. tarda* vaccine in turbot [39]. Considering the bactericidal properties of lysozyme and the increased activity of serum lysozyme in the groups immunized with the vaccine compared to the control group, it probably indicates the greater function of the non-specific immune system of fish against pathogenic agents.

Serum antibody titer is one of the most vital indicators to evaluate the immunogenicity of vaccines [38]. The comparison of serum antibody titers against *S. iniae* (in the SI, VI, SVI and SVIM groups) and *V. harveyi* (in the SI, VI, SVI, VIM and SVIM groups) in the vaccinated groups was significant compared to the control group. In line with our results, the bivalent *A. salmonicida* and *V. anguillarum* vaccine in Atlantic salmon [40], the polyvalent yersiniosis and streptococcosis/lactococcosis vaccine in *O. mykiss* [6], the bivalent vibriosis and edwardsillosis vaccine in turbot [38], the bivalent vibriosis vaccine in turbot [41], the bivalent vibriosis in gilthead sea bream [42] and the bivalent *V. scophthalmi* and *A. salmonicida* vaccine in turbot [34], support our findings. The reason for the increase in the levels of antibodies may be due to the specific production by the host. Meanwhile, the cell membrane of pathogenic organisms is in direct contact with the host’s immune system, and it has been proven that the membrane molecules of different types of microorganisms can stimulate strong cellular and humoral immune responses in fish. Effective mechanisms in the production of specific antibodies against proteins may include eliminating the ability of bacteria to stick and attack the host tissue, opsonizing bacterial cells and stimulating phagocytosis by macrophages and activating the complement cascade [43].

By evaluating the RPS, it is possible to check the resistance of the fish against the disease and the immunogenicity of the vaccine [41]. The RPS after exposure to *S. iniae* (SI, VI and SVI) and *V. harveyi* (VI, SVI, VIM and SVIM) had a significant increase compared to the control group. Several reports are showing that vaccination has increased RPS in fish (6, 14, 49). Similar to our findings, the RPS in the groups affected by polyvalent bacteria (*V. parahaemolyticus* and *V. alginolyticus*) and (*V. parahaemolyticus* and *V. alginolyticus*) were reported as 91.75% and 75%, respectively [42]. In another study, 80% (*S. iniae*), 90% (*L. garvieae*) and 80% (*Y. ruckeri*) RPS in the group immunized with injection vaccine and 50% (*S. iniae*), 70% (*L. garvieae*) and 60% (*Y. ruckeri*) were observed in the group immunized with immersion vaccine compared to the control group [6]. Also, in Asian seabass compared to the control group in line with our results, RPS after challenge with *V. harveyi*, *A. hydrophila*, and *S. agalactiae* was reported as 75%, 80%, and 80%, respectively (14). In another study, the percentage of relative survival after 14 days of challenge with *S. iniae* (70%), *L. garviae* (60%) and *Y. ruckeri* (76.6%) in the group immunized with injectable vaccine was significant compared to the control group (*P* < 0.05) [44].

## Conclusion

Immunization using bivalent and polyvalent vaccines is one of the most ideal ways to deal with diseases, increase stimulation of the immune system response and reduce the cost of vaccination in farmed fish. Additionally, immunization of fish with inactivated vaccines via injection method, is a good and effective strategy to control many aquaculture diseases, since it provides a strong immune response, reduces infection rate, and increases survival rate. In general, the results showed that the bivalent formalin inactivated (*S. iniae* and *V. harveyi* ) vaccines had a significant increase in immunological parameters, antibody titer and RPS. Meanwhile, vaccination using bivalent vaccines by injection method has better immunogenicity than monovalent immersion and injection vaccines.

## Materials and methods

### Vaccine preparation

For this research, a pathogenic *S. iniae* (9609NB) and *V. harveyi* (SB9612N4) bacterial strains that were previously isolated from diseased Asian seabass cage cultures in Bushehr province (north of Persian Gulf, Iran) were used. These pathogenic strains, *S. iniae* in blood agar and *V. harveyi* in thiosulphate-citrate-bile-salts-sucrose (TCBS) agar, were cultured and maintained (30 ˚C for 48 h). Then bacterial strains were transferred to tryptone soy broth (TSB) medium (with the addition of NaCl 1.5% w/v for *V. harveyi* and *S. iniae*) at 30 ˚C for 48 h. Bacterial suspensions (*S. iniae* and *V. harveyi*) were mixed with 1% formalin and kept at 4 °C for 24 h. Afterward, the formalin inactivated bacterial mixture were centrifuged at 6000 × g for 10 min at 4 °C, the supernatant were discarded and the bacterial pellets were washed three times with sterile PBS. (To remove the remaining formalin). The bacterial pellets resuspended after washing in equal volumes of PBS to 10^10^ CFU/mL. The concentration of *V. harveyi* and *S. iniae* was adjusted to 10^10^ CFU/mL bacteria. Finally, the formalin killed bacterial suspensions (bacterin) were kept at 4 °C until use [42].

### In-vivo experimental design

Asian seabass (*L. calcarifer*), (1200 pieces, 132.6 ± 25.4 g) was purchased from Bushehr, Iran and stored in breeding tanks. The fish were kept for two weeks to adapt before the investigation. Then, the fish were randomly divided into eight treatments in triplicate (500-liter tanks, 50 fish in each tank). Bivalent inactivated (*S. iniae* and *V. harveyi*) and monovalent inactivated (*S. iniae* and *V. harveyi*) vaccines were used for Asian seabass vaccination after stopping feeding for two days. The treatments are described in Table 1. The commercial feed (21 Beyza Mill Co., Shiraz, Iran) containing 46% crude protein, 16% crude fat, 3% fiber, 12% ash, 10% moisture and 4300 kcal/kg of digestible energy was used as the basic ration. The fish were hand-fed to apparent satiation twice a day. The physico-chemical factors of water such as dissolved oxygen (6.93 ± 0.25 mg/L^− 1^), water temperature (30 ± 1 °C), salinity (28 ppt) and photoperiod (12 h light: 12 h dark) were maintained during the acclimation and experimental period.

**Table 1:** Experimental design

Fish number in each group	Immunization method	Type of vaccine	Group name
150	Injection	*S. iniae*	SI
150	Injection	*V. harveyi*	VI
150	Injection	Bivalent *S. iniae* and *V. harvey*	SVI
150	Immersion	*S. iniae*	SIM
150	Immersion	*V. harveyi*	VIM
150	Immersion	Bivalent *S. iniae* and *V. harvey*	SVIM
150	Injection	Phosphate-buffered saline	PBSI
150	-	Without vaccine administration	CTRL

### Vaccine administration

Monovalent and bivalent inactivated (*S. iniae* and *V. harveyi*) vaccines were used respectively to immunize fish. For immersion method, fish were immunized (SIM, VIM, and SVIM) in 10^9^ CFU/mL vaccine (1:10 dilution of vaccine stock) for two minutes with adequate aeration. Following the same order of the experimental groups in Table 1, for the injection method, after anesthetizing the fish with clove powder (75 mg/L^− 1^), 0.1 mL of vaccine was injected intraperitoneally (IP) into the fish (SI, VI, and SVI). The PBSI group was injected with 0.1 mL of PBS instead of the vaccine.

### Sampling procedure and measurement of hematological parameters

On the 30th day and 60th day after vaccination, the fish were stopped feeding for 24 h [42, 49]. Then the fish were anesthetized with clove powder (75 mg/L^− 1^), and blood samples were taken from the caudal vein (9 fish from each treatment). Blood samples (2 mL) were divided for serum separation (without anti-coagulant) and for measuring hematological parameters (containing heparin anti-coagulant). The blood samples without anti-coagulant were centrifuged at 3500 rpm for 10 min at room temperature (RT). Then supernatants (serum) were kept at − 80 °C for further analysis. Following the methodology described by Grant for measuring blood parameters [45]. In each stage of sampling, 9 fish were sampled from each treatment.

### Serum biochemical parameters

Biochemical parameters of blood serum such as total protein (Tp) and albumin (Alb) were determined using a commercial kit (Pars Azmoon, Tehran, Iran) based on the methodology of Hoseinifar et al. [46]. The level of serum globulin (Glb) was estimated by subtracting the amount of Alb from the amount of Tp [46].

### Measurement of non-specific immune parameters

Respiratory burst activity was done using Yonar method with some modification [47]. To measure the respiratory burst activity of WBCs, 0.1 mL of heparinized blood was mixed with 0.1 mL of 0.2% nitro blue tetrazolium (NBT) solution and incubated for 30 min at 25 °C. Then 0.1 mL of the mixture was added to 1 ml of dimethylformamide solution. After centrifuging the samples, the supernatant was measured at a wavelength (WL) of 540 nm [47]. The serum lysozyme activity was measured by enzyme-linked immunosorbent assay (ELISA) method. Hence, 9 mg of *Micrococcus luteus* bacterial wall was dissolved in 30 ml of sodium phosphate buffer (pH = 6.2). Then, 10 µL of serum sample was mixed with 90 µl of bacterial wall suspension. The samples were recorded with a WL of 450 nm for 10 min. Each 0.001 unit of decrease in absorbance per minute was considered equivalent to one active unit of lysozyme [48]. To measure complement activity, inactive serum of rabbits immunized with sheep RBCs at a concentration of 25% with 10% sheep RBCs was stored for 24 h at 4 °C and washed with PBS and the supernatant was discarded. Then, 25 µL of active sera plus 375 µL of complement buffer and 100 µL of 5% washed sheep red blood cells were mixed and placed in a 37 °C incubator for 45 min. The mixed solution was centrifuged for 5 min at 3,000 g per minute and 100 µL of the supernatant of each serum was collected and poured into a 96-well ELISA plate, and finally, the optical density (OD) of the samples was measured at a WL of 490 nm [49].

### Bactericidal activity

To measure the bactericidal activity of serum, the method described by Budino et al. [50] was used with some modifications. Briefly, after *V. harveyi* (2 × 10^7^ CFU/mL) was added to the serum samples, they were incubated for 6 h at 20 °C and then MTT (dimethylthiazole-diphenyltetrazolium bromide) was added to the suspension. After 15 min of incubation at RT, the samples were measured by the color change caused by the reduction of MTT by living bacteria at a WL of 630 nm by an ELISA reader.

### Antibody titer

Serum antibody titer against *S. iniae* and *V. harveyi* was measured by the method recommended by Skov et al. [51] with slight modifications by the ELISA reader. Briefly, 50 µL of soluble antigens [*S. iniae* and *V. harveyi* (100 g/mL^− 1^)] in bicarbonate buffer (pH = 9.6) were added to each well of the ELISA plates and incubated overnight at 4 °C. The samples of each well were washed three times with 1% PBS containing Tween-20 (PBS-T). Then, they were blocked with 2.5% skim milk in PBS-T for 3 h at 37 °C. After 3 washes with buffer, the serum samples were diluted 1:400 in PBS-T and 1% skim milk and 100 µL of the suspension was added to the wells. The plate was placed on a shaker for 90 min at RT and after washing 4 times, the rabbit’s anti-seabass polyclonal antibody was diluted 1:20. 100 µL of the suspension was added to the wells and incubated for one hour at RT. After washing, 50 µL of goat anti-rabbit IgG HRP conjugate with a dilution of 1:3000 was added to the wells. Finally, 50 µL of chromogenic substrate solution was added to the wells and after 10 min of incubation at RT, 50 µL of stopping solution (2% sulfuric acid) was added to the wells. The OD of the samples was determined by an ELISA reader at a WL of 490 nm.

### Determination of lethal dose (LD_50_)

To determine the LD_50_, 10-fold serial dilutions (10^5^-10^8^ CFU mL^− 1^) using PBS were prepared for *S. iniae* and *V. harveyi*. 0.1 mL of each bacterial dilution was injected IP into 10 fish per each bacterial strain, separately. After IP injection, mortality was recorded for 10 days and then the LD_50_ was determined with Probit software.

### Challenge experiment

To determine the percentage of survival rate (SR) after 60 days of vaccination, 30 fish from the group (10 fish from each replication) were challenged IP and observed for 14 days. All fish groups were challenged against each individual bacterium (*S. iniae* and *V. harveyi*) to detect the cross-protection level. Prior to injection, fish were anesthetized with clove powder (75 mg/L^− 1^), then injected with 0.1 mL of each bacterial suspension (LD_50_ = 4.9 × 10^6^ CFU mL^− 1^ of *S. iniae* and LD_50_ = 6.8 × 10^7^ CFU mL^− 1^ of *V. harveyi*); separately were injected IP to the fish. The cumulative mortality of fish was recorded for 14 days and the relative percentage of survival (RPS) of fish was calculated through the following formula [14].

$${\rm{RPS = 1 - }}\left( {{\rm{mortality}}\,\,{\rm{in}}\,\,{\rm{vaccinates}}\,{\rm{/}}\,{\rm{mortality}}\,{\rm{in}}\,{\rm{controls}}} \right){\rm{ \times 100}}$$

After the bacterial challenge, and at the end of the experiment, the fish were treated and healthy fish returned to rearing conditions.

### Statistical analysis

The GraphPad PRISM software (version 9.5.1.733) was used for statistical data analysis. First, the homogeneity and normality of the data were checked using the Kolmogorov-Smirnov statistical test. Then, two-way analysis of variance (Two-way ANOVA) and Tukey’s analysis of variance were performed to compare vaccinated and non-vaccinated groups over time. One-way analysis of variance (One-way ANOVA) and Tukey’s test were used to analyze survival rate data using GraphPad Prism software. Data are presented as mean ± SD. Significance level was set at *P* < 0.05.

#### Accession numbers

*S. iniae* (accession number 9609NB) and *V. harveyi* (accession number SB9612N4).

## Data Availability

No datasets were generated or analysed during the current study.

## Abbreviations

*S. iniae*: *Streptococcus iniae*
*V. harveyi*: *Vibrio harveyi*
SI: *Streptococcus iniae* injection
VI: *Vibrio harveyi* injection
SVI: bivalent *S. iniae* and *V. harvey* injection
SIM: *Streptococcus iniae* immersion
VIM: *Vibrio harveyi* immersion
SVIM: bivalent *S. iniae* and *V. harvey* immersion
CTRL: Control
Tp: total protein
Alb: albumin
Glb: globulin
RPS: relative percentage of survival
PTCC: Persian type culture collection
SD: standard deviation
LD: lethal dose
ANOVA: analysis of variance
SR: survival rate
RT: room temperature
OD: optical density
ELISA: enzyme-linked immunosorbent assay
